# Primary inflammatory myofibroblastic tumor of the stomach in an adult woman: a case report and review of the literature

**DOI:** 10.1186/1477-7819-11-35

**Published:** 2013-02-04

**Authors:** Milos Bjelovic, Marjan Micev, Bratislav Spica, Tamara Babic, Dragan Gunjic, Aleksandra Djuric, Predrag Pesko

**Affiliations:** 1 Faculty of Medicine, University of Belgrade, Belgrade, Serbia; 2 Clinic for Digestive Surgery – (First Surgical Clinic), Clinical Center of Serbia, Belgrade, Serbia

**Keywords:** Stomach, Inflammatory myofibroblastic tumor, Inflammatory pseudotumor, Dyspepsia, Surgery, Adults

## Abstract

Inflammatory myofibroblastic tumor has been defined as a histologically distinctive lesion with uncertain behaviour. The term inflammatory myofibroblastic tumor more commonly referred to as “pseudostumor ”, denotes a pseudosarcomatous inflammatory lesion that contains spindle cells, myofibroblasts, plasma cells, lymphocytes and histiocytes. It exhibits a variable biological behavior that ranges from frequently benign lesions to more aggressive variants. Inflammatory myofibroblastic tumor mostly occurs in the soft tissue of children and young adults, and the lungs are the most commonly affected site, but it has been recognized that any anatomic localization can be involved. Inflammatory myofibroblastic tumors in adults are very rare, especially in the stomach. We present a case of a 43-year old woman with primary inflammatory myofibiroblastic tumor in the stomach and a review of the literature.

## Background

Inflammatory myofibroblastic tumor (IMT) has been defined as a histologically distinctive lesion of uncertain behavior. It has also been previously known as inflammatory pseudotumor, plasma cell granuloma, inflammatory myofibroblastoma, and inflammatory myofibrohistiocytic proliferation. IMT frequently recurs and rarely metastasizes [1].

It seems that a large proportion of these lesions are true mesenchymal neoplasms and the term IMT more commonly referred to as “pseudotumor”, refers to a pseudosarcomatous inflammatory lesion that mostly occurs in the soft tissue of children and young adults, and contains spindle cells, myofibroblasts, plasma cells, lymphocytes and histiocytes [2,3]. The pathogenesis of IMT remains unclear, although various allergic, immunologic, and infectious mechanisms have been postulated [4]. IMT rarely occurs in adult persons, particularly in the stomach [2,3]. The previously described cases of primary gastric IMTs were in the form of case reports or small series [5-8]. Here we present the case of a primary gastric IMT in an adult woman and a review of the literature.

## Case presentation

**Figure 1:**
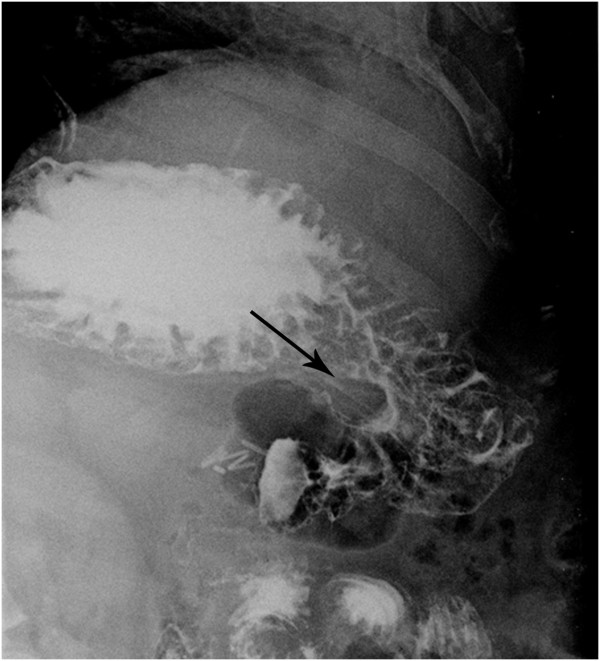
**Double-contrast barium meal of the stomach showing the inflammatory myofibroblastic tumor (IMT). **A round, sharply contoured filling defect was identified, situated on the lesser curvature of the stomach, near the angular incisure, approximately 2.5 cm in diameter.

**Figure 2:**
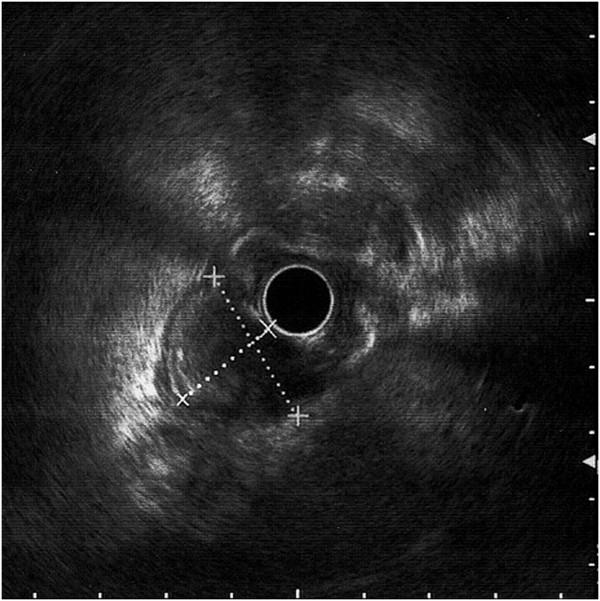
**Endoscopic ultrasound (EUS) of the inflammatory myofibroblastic tumor (IMT). **An oval hypoechoic mass, 25 × 17 mm in diameters was identified arising from the muscularis propria layer.

**Figure 3:**
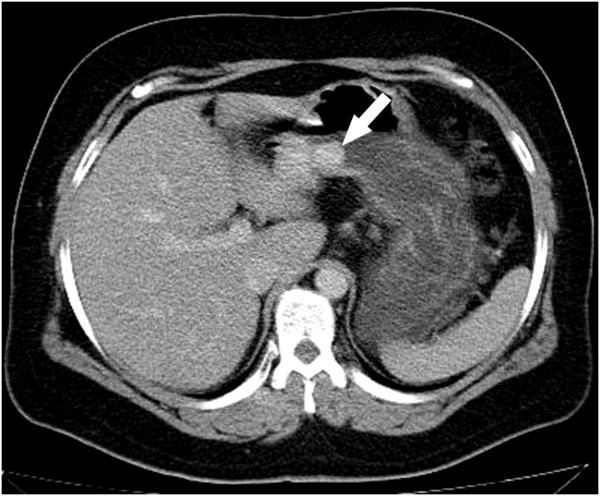
**Abdominal computed tomography (CT) scan of the stomach showing the inflammatory myofibroblastic tumor (IMT). **Hyperattenuating, focal, polypoid posterior wall thickening was identified near the angular incisure of the stomach.

**Figure 4:**
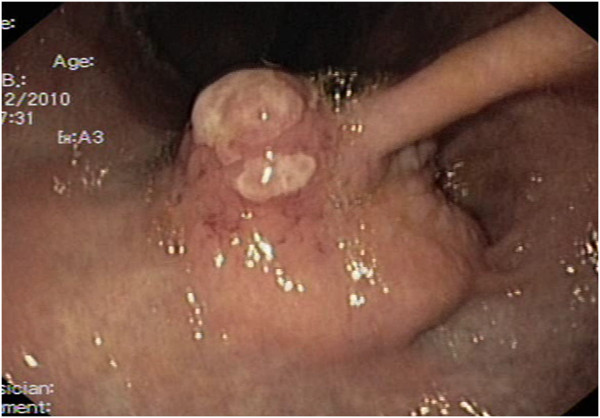
**Upper flexible endoscopy of the inflammatory myofibroblastic tumor (IMT). **A round, white-gray, sessile and hard lesion was identified, with surrounding, distal, flattening infiltration of the gastric wall. The lesion was partially covered with fibrin, and was localized below the lesser curvature in the distal part of the stomach.

A 43-year old caucasian woman was presented with gastrointestinal symptoms e.g. epigastric pain, nausea and pyrosis. She had had gastrointestinal symptoms for 2 years, a positive medical history of a c-section for a year, and a laparoscopic cholecystectomy 2 years before this consultation. On admission her body mass index (BMI) was 37,2 kg/m^2^, and she had Karnofsky score of 100%. Physical examination showed mild abdominal tenderness in the epigastric region, but no palpable abdominal mass. Her laboratory analyses were unremarkable. The chest X-ray film was normal. Round, sharply contoured filling defect, situated on the lesser curvature of the stomach, near the angular incisure, approximately 2.5 cm in diameter, was demonstrated by a double-contrast barium meal (Figure 1). Endoscopic ultrasound (EUS) showed an oval hypoechoic mass, 25 × 17 mm in diameter, arising from the muscularis propria layer (Figure 2). Abdominal computed tomography (CT) demonstrated the hyperattenuating focal polypoid posterior wall thickening of the gastric angle, with no evidence of adjacent organs infiltration or metastatic disease (Figure 3). Upper flexible endoscopy demonstrated a round, white-gray, sessile and hard lesion, with surrounding distal flattening infiltration of the gastric wall. Lesion was in a part covered with fibrin, and localized on the lesser curvature below the angular incisure (Figure 4). There was no active, or any sign of recent bleeding. Multiple endoscopic biopsy samples were taken. Histopathological evaluation of the biopsy samples demonstrated unclear hypercelullar proliferation consisting of mesenchymal and inflammatory cells. However, focal atypia gave rise to suspicion of sarcomatous or even anaplastic cell neoplasia with unclear histogenesis.

**Figure 5:**
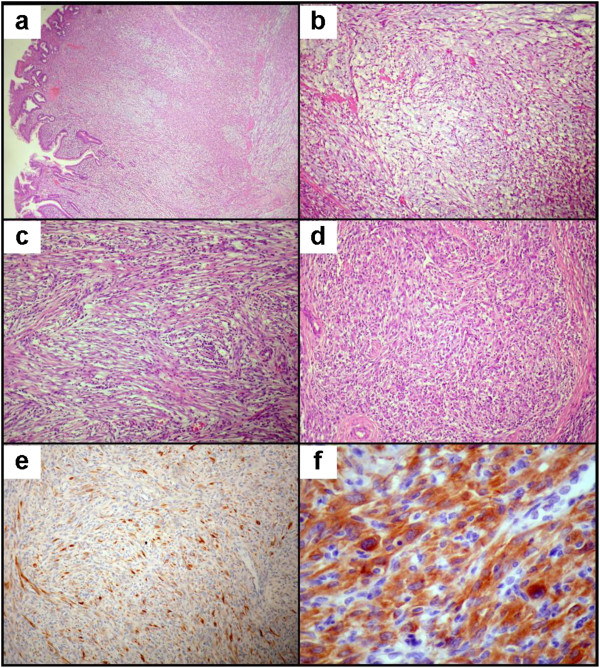
**Findings on histology of gastric inflammatory myofibroblastic tumor (IMT). **(**a**) Mucosal and transmural infiltration of mesenchymal and inflammatory cells with (**b**) focal mucoid change. (**c**) Characteristic loosely organized fascicles of myofibroblastic cells admixed with diffuse inflammatory infiltration and (**d**) focal mixed epithelioid and spindle cell presentation. Immunohistochemistry showed (**e**) sparse desmin immunoexpression and (**f**) diffuse strong cytoplasmic anaplastic lymphoma kinase (ALK) protein immunoexpression with some perinuclear intensification.

Due to gastric tumor with unclear tumor histology, surgical excision was suggested. The patient underwent an exploratory laparotomy. During surgery tumor formation was found in the distal part of the stomach closer to small curvature. The tumor mass involved the entire thickness of the gastric wall. There were no signs of adjacent organs infiltration or metastatic disease. Subtotal gastrectomy with D2 lymph node dissection and Roux-en-Y reconstruction was performed. Early postoperative course was uneventful and patient was discharged from the hospital 8 days following the surgery. Macroscopic examination of the resected portion of the stomach revealed a well demarcated tumor mass in the distal part of the stomach, 60 × 40 mm in diameter, with superficial polypoid protrusion, which on cut surface resembled an ill defined whitish nodular fraction measuring 25 × 17 × 17 mm. Histologically, there was an ill-defined proliferation composed mostly of spindle-shaped mesenchymal cells admixed with diffusely scattered inflammatory cells. The mitotic count was 1/10 high power fields (HPF), and Ki-67 labeling index was estimated at 8%. Cellular atypia was focal and seemed reactive, as mostly observed in the vicinity of ulcerative superficial portions. There was no tumor necrosis. Lymph nodes found in the perigastric region and celiac trunk all tested negative for tumor. Contrary to the inflammatory fibroid polyp, the lesion had less eosinophils and fibroses but more lymphoid cell infiltrates. Moreover, a regular vascular pattern or “perivascular cuffing” was not observed, which is regarded as a feature of inflammatory fibroid polyps, rather than inflammatory myofibroblastic tumors. On immunohistochemistry, tumor cells showed positive immunoreactivity for vimentin, although only focal smooth muscle actin (SMA) and sparse desmin immunoexpression, while remainnig negative for pan-cytoceratin, c-kit, CD 34 and S-100. These findings and evident immunoexpression of anaplastic lymphoma kinase (ALK) protein were consistent with an IMT originating from the gastric wall. Having in mind the positive immunoreactivity for ALK and on consulting referent data it was concluded that the particular IMT might have recurrent potential and possible aggressive prognosis, thus representing uncertain metastatic biological behavior (Figure 5).

The patient was without symptoms over 24 months of follow-up. During this period the US, CT and upper flexible endoscopy showed no signs of tumor recurrence or metastatic disease.

## Conclusion

IMT is a rare lesion, considered to be morphologic expression of reactive, reparative, infective and neoplastic processes [9]. There has been debate as to whether IMT is a tumor or inflammation, and also whether is benign or malignant. It is locally recurrent, however it rarely metastasizes [1]. IMT is histopathologically composed of myofibroblastic spindle cells with inflammatory cell infiltrate of plasma cells, lymphocytes and eosinophiles [1]. It is mainly seen in children and young adults, and the lungs are the most commonly affected site, but it has been recognized that any anatomic localization can be involved. The first abdominal localization was described in liver by Pack and Baker in 1953 [10]. Primary gastric IMT in adults are very rare [5].

We presented the case of a 43-year old caucasian woman with prior medical history of a c-section a year, as well as a laparoscopic cholecystectomy two years prior to this medical consultation. It has been suggested that IMT is a reactive, reparative lesion which occurs after trauma or surgery (Table 1), [6,11].

**Table 1:** Summarized clinicopathological characteristics of previously reported primary gastric IMT

**Author**	**Sex/Age**	**Presenting symptoms**	**Tumor localization in the stomach**	**Tumor size (in cm)**	**Mitosis (10 HPFs)**	Histologic pattern*	**Treatment**	**Follow-up**
**Shi et al.**[5]	M/36	AP, AM	Antrum, LC	4.5 cm	1-2	Myxoid hypocellular with some fascicular areas	PG	NED, 5 years
**Shi et al.**[5]	M/42	AP,UGH, AM	Upper body, GC	8.0 cm	1-2, focally up to 5	Fascicular with some myxoid areas	PG	Recurrence at 12 months after the first surgery, now NED at 2 years (11 month after the second surgery)
**Shi et al.**[5]	M/40	AM	Upper body, AW	6.3 cm	1-2	Myxoid hypocellular with some fascicular areas	PG	NED 3.3 years
**Shi et al.**[5]	M/45	AP, AM	Angle	5.5 cm	1-2	Myxoid hypocellular with some fascicular areas	PG	NED, 2.6 years
**Shi et al.**[5]	W/40	AP, AM	Lower body, PW	5.8 cm	1-2	Fascicular with some myxoid and sclerotic areas	PG	NED, 4 years
**Albaryak et al.**[7]	W/56	UGH, nausea, vomiting	C extending towards P	11 cm	1-2	Granulation-type and storiform spindle cell proliferation	PG	NED, 8 months
**Leon et al.**[3]	W/50	Vomiting, early satiety, weight loss	PW	7 cm	1-2	Patternless round and spindle cell proliferation	PG	NED,2 years
**Park et al.**[8]	W/55	AP, hematoperitoneum	Upper body, AW near GC	8 cm	1-2	Vague fascicular proliferation	Gastric wedge resection	/
**Bjelovic et al.**	W/43	AP, pyrosis, nausea	Distal stomach, below AI	6 cm	1-2	Hypercellular spindle cell proliferation with vague fascicular areas	DG	2 years

AP, abdominal pain; AM, abdominal mass; UGH, upper gastrointestinal hemorrhage, LC, lesser curvature of the stomach; GC, greater curvature of the stomach; AW, anterior wall of the stomach; PW, posterior wall of the stomach; C, cardia; AI, angular incisure of the stomach; PG, partial gastrectomy; DG, distal gastrectomy; NED, no evidence of disease.

In this case, due to relatively small tumor, patient had dyspeptic symptoms for two years. However, IMT clinical presentation varies depending on its anatomical localization in the stomach and size (Table 1).

Even with a thorough diagnostic workup, which included CT, US, EUS, laboratory analyses, upper flexible endoscopy with biopsy in this case, it was difficult to make an accurate preoperative tumor diagnosis. In most cases IMT features mimic malignancy on upper flexible endoscopy and radiological imaging. For that reason, most IMT cases require surgical exploration and complete resection to obtain an accurate microscopic diagnosis, [6].

Due to a good general condition and no signs of metastatic disease, a radical surgical procedure was performed including a subtotal gastrectomy with D2 lymph node dissection and Roux-en-Y reconstruction. A complete surgical resection remains the only proven mode of cure, and is proposed as the first line of treatment in all IMT cases [12]. Types of surgical procedures used in other cases of primary gastric IMTs depended on tumor localization and size as well as general condition of patients (Table 1).

In the given case, pathology revealed a clearly demarcated tumor mass located in the distal part of the stomach which involved full thickness of the gastric wall, without any signs of adjacent organs infiltration. The lymph nodes were all negative for tumor. The histological features of the tumor included myofibroblastic proliferation and a varying degree of inflammatory cells mainly consisting of lymphocytes, histiocytes and plasma cells. Similar histological patterns were described in other cases of primary gastric IMTs, (Table 1).

In this case the tumor cells showed a positive immunnoreactivity with ALK protein. The presence of ALK protein expression essentially serves as a surrogate marker for the translocation involving the ALK locus at chromosome 2p23, hence excluding a reactive myofibroblastic proliferation. These results were suggestive of IMT with possible aggressive prognosis and metastatic potential. However, recently published data suggest that ALK protein expression and epitheliod or round cell morphology rather than other pathologic features correlate well with the behavior. For those cases, the term epithelioid inflammatory myofibroblastic sarcoma has been proposed [13].

Within a year of a surgery, 15% to 37% cases of primary gastric IMT ended with tumor recurrence [14]. In every primary gastric IMT case a long- term clinical, radiological and laboratory follow-up was indicated. In the presented case after 24 months of follow-up period the patient was symptoms free, with no signs of tumor recurrence or metastatic disease.

## Consent

Written informed consent was obtained from the patient for publication of this Case report and any accompanying images. A copy of the written consent is available for review by the Editor-in-Chief of this journal.

## Abbreviations

ALK: Anaplastic lymphoma kinase; BMI: Body mass index; CT: Computed tomography; EUS: Endoscopic ultrasound; HPF: High power field; IMT: Inflammatory myofibroblastic tumor; SMA: Smooth muscle actin; US: Ultrasound.

## Competing interests

The authors declare that they have no financial and non-financial competing interests.

## Authors’ information

Name and Surname: **Milos M. Bjelovic MD, Ph.D.**

Date and Place of Birth December 23^rd^, 1963; Belgrade, Serbia

Academic Degrees University of Belgrade, Ph.D. 2003.

Research: “Role of the splenectomy and dissection of lymph nodes along the upper border of the pancreas in the radial surgical treatment of gastric cancer”

Faculty Appointments University of Belgrade, School of Medicine Professor of Surgery 2012.

Hospital Appointments The First University Surgical Hospital, Belgrade

Head of the Department for Minimally Invasive Upper Digestive Surgery and Assistant Director of the Hospital 2010.

Education abroad Digestive and Oncologic Surgery, National Cancer Center Hospital, Tokyo and Tokushukai Medical Group, Japan 2002.

Bariatric Surgery, Dr Miler, Salzburg, Austria 2004.and 2010.

Training course in advanced laparoscopic procedures, Center for the Experimental and Microsurgery, University “Victor Babas” Temisuara, Romania 2004.

Minimal Invasive Surgery of the Esophagus, Prof. James Luketich, Department of Cardiothoracic Surgery, UPMC, Pittsburgh, PA, USA 2007. and 2009.

Minimal Invasive Surgery of the Incisional and Umbilical Hernias, Dr Tim Tollens, Mehelen, Denmark 2009.

LEES Training Course, Olympus Training Center Hamburg, Germany 2010.

Master Course TME, Covidien Training Center Erlancourt, France 2011.

Single Incision and Reduced Port Surgery Training Course, Vienna, Austria 2011.

## Authors’ contributions

MB, TB contributed equally to this work. MB, BS and TB designed reasearch. MB, BS, TB, DG, performed reasearch. AD analyzed radiografic images. MM performed pathohistological and immunohistochemistry evaluation. BT wrote the paper. MB, MM, PP revised article for important intelectual content. MB, MM, PP gave final approval for version to be published. All authors read and approved the final manuscript.
